# Visual Performance of Defocus Incorporated Multiple Segments With Triple Enhanced Design Spectacle Lenses Versus DIMS and Single Vision Lenses

**DOI:** 10.1167/tvst.15.7.32

**Published:** 2026-07-29

**Authors:** Carly Siu Yin Lam, Kenneth Ka King Liu, Han Yu Zhang, Paul Lee, Hua Qi, Keigo Hasegawa, Shohei Matsuoka, Daisy Ka Yan Leung, Dennis Yan Yin Tse, Chi Ho To

**Affiliations:** 1 Centre for Myopia Research, School of Optometry, The Hong Kong Polytechnic University, Hong Kong, China; 2 School of Medicine, Nankai University, Tianjin, China; 3 Southampton Clinical Trials Unit, University of Southampton, Southampton, UK; 4 Technical Research and Development, HOYA Vision Care, HOYA Corporation, Tokyo, Japan; 5 Centre for Eye and Vision Research Limited, Hong Kong

**Keywords:** myopia, myopic defocus, myopia control, spectacle lens

## Abstract

**Purpose:**

To compare short-term visual functions changes in myopic children who had worn the defocus incorporated multiple segment (DIMS), DIMS with triple enhanced design (DIMS TED), and single-vision (SV) spectacle lenses.

**Methods:**

Thirty-eight Chinese children (six to 13 years) with spherical equivalent refractive error −1.00 D to −5.00 D were recruited. Participants wore DIMS, DIMS TED and SV spectacle lenses in random order. High- and low-contrast distance and near visual acuity (VA), mid-peripheral near VA, binocular functions, accommodation and visual symptoms were assessed before and after 1-week of lens wear, while mid-peripheral distance VA was assessed with and without glare in a second visit.

**Results:**

Most visual functions showed no differences before and after lens wear. Central high- and low-contrast distance VA and central high-contrast near VA under room and dim lighting were unaffected by DIMS and DIMS TED spectacle lenses compared to SV lenses (all *P* > 0.05). Central low-contrast near VA was reduced by 0.06 ± 0.10 logMAR in DIMS TED lenses compared to DIMS lenses under room lighting (*P* < 0.01), and by 0.06 ± 0.11 logMAR compared with SV spectacle lenses under dim lighting (*P* < 0.01). Mid-peripheral distance VA and near VA were reduced by approximately 0.10 logMAR in DIMS TED and DIMS compared to SV lenses (*P* < 0.01), respectively. However, these reductions are unlikely to represent a clinically meaningful impairment in visual function. Most children adapted and accepted the DIMS TED spectacle lenses well.

**Conclusions:**

DIMS TED spectacle lenses demonstrated visual performance comparable to DIMS and SV spectacle lenses.

**Translational Relevance:**

The enhanced design of the DIMS TED spectacle lenses maintains visual performance comparable to DIMS and single vision spectacle lenses, and may potentially increase myopia control efficacy.

## Introduction

Myopia, or near-sightedness, is one of the significant public health concerns in the world because it may increase the risks of the ocular complications such as glaucoma, cataract and retinal detachment.^1^ The rising prevalence of myopia^2^ has increased interest in strategies to delay onset and slow progression to reduce ocular complications.

Animal studies have provided strong evidence that imposed myopic defocus inhibits eye growth while hyperopic defocus promotes eye growth.^3^ Studies using chicks,^4^^–^^5^ guinea pigs^6^ and rhesus monkeys^7^ showed myopic eye growth could be retarded or reversed by using dual-powered or multifocal lenses. The peripheral visual signals were found to be crucial for central refractive development and the effect of peripheral myopia defocus can provide myopia control signals.^8^^–^^9^ There are different clinical interventions to slow the myopia progression in children, including atropine, orthokeratology lenses, multifocal soft contact lenses, progressive addition spectacles and peripheral defocus spectacle lenses.^10^^–^^12^ Each intervention has its own advantages and side effects. Among these interventions, myopia control spectacles may be considered as one of the first approaches to combat the myopia progression because of its minimal side effects. Utilising the myopic defocus theory, DIMS spectacle lens (DIMS is commercially marketed as MiYOSMART spectacle lens by HOYA, Japan) was designed with a clear central optical zone for correcting refractive errors and surrounded by multiple segments of constant myopic defocus (+3.5D) at the mid-periphery, providing clear central vision and peripheral myopic defocus simultaneously. A 24-month randomized controlled clinical trial on myopia control using DIMS spectacles lenses demonstrated 52% efficacy in slowing myopia progression and 62% in slowing axial elongation when compared with the control group of children who wore single vision spectacle lenses.^13^ And the myopia control effects by DIMS spectacle lenses were sustained over 6 years.^14^

Previous studies suggested that increased the amount of defocus power generally enhanced myopia control efficacy including the use of multifocal soft contact lenses^15^^–^^16^ and peripheral defocus spectacles.^17^ For example, Zhang et al.^16^ modified the Defocus Incorporated Soft Contact (DISC) lens to a stronger primary myopic defocus (from +2.50 D to +3.50 D), with peripheral rings increasing up to +6.00D. The modified DISC3.5plus contact lens demonstrated significant reduction in axial elongation and myopia progression compared to single vision contact lenses. The DIMS triple enhanced design (TED) spectacle lens is designed to aim at enhancing myopia control efficacy by minimizing the central clear zone, increasing the area ratio of correction and myopic defocus zones, and applying greater myopic defocus power. However, with the increased defocus power, the visual quality may be compromised which may lead to poor compliance. The current study aims to evaluate and compare short-term visual performance between the newly developed DIMS TED spectacle lenses, DIMS spectacle lenses, and single-vision (SV) spectacle lenses.

## Material and Methods

### Subjects

Children aged six to 13 were recruited for this clinical study as they are the majority population to benefit from the myopia control spectacle lenses The inclusion criteria were as follows: (1) Spherical equivalent refraction: −1.00 to −6.00 Dioptres (D); (2) Astigmatism and anisometropia of 1.50 D or less; (3) Monocular best-corrected VA is equal to or better than 0.00 logarithm of minimal angle of resolution (logMAR); (4) Monocular best-corrected VA with cycloplegic refraction equal to or better than 0.00 logMAR; (5) Willingness to wear the study spectacles regularly; (6) Acceptance of the masked study design; (7) No ocular pathology or former history of myopia control treatment.

Thirty-eight myopic schoolchildren (18 males and 20 females 10.5 ± 1.8 years of age; SER −2.63 ± 1.09 D) were enrolled for Part 1 (Measurement of Central Distance and Near VA and Mid-Peripheral Near Visual Acuity VA Under Different Luminance Levels as Well as Binocular Functions) and Part 2 of the study (Measurement of Mid-Peripheral Distance VA Under Different Luminance Levels).

All procedures of the study followed the tenets of the Declaration of Helsinki and were approved by the Human Subjects Ethics Committee of The Hong Kong Polytechnic University Institutional Review Board (Ref no.: HSEARS20211008007-04). Written informed consent from parents and assent from children were obtained before the study.

### Study Design

Three types of spectacle lenses were used in the study, the single vision (SV) spectacle lens, the DIMS spectacle lens, and the DIMS TED spectacle lens. After comprehensive ocular health examination, cycloplegic eye drops (one drop of proparacine 0.5% and then one drop of cyclopentolate HCL 1%) were instilled into the eyes and tested to confirm accommodation is relaxed. Subjective refraction with maximum plus to maximum VA was done to determine the prescription. The spectacle lenses were ordered according to the cycloplegic subjective refraction. Once the spectacles were ready, participants were randomly assigned to wear one of the three spectacle lens types, with visual performance assessed at the same visit. After one week, participants returned for repeat testing, and the procedure was repeated for the remaining type of spectacle lenses, each worn for one week followed by assessment. A pregenerated random sequence of the three-testing lenses (DIMS, DIMS TED, SV lenses) was created before recruitment began. The sequence was concealed from all investigators who participated in data collection throughout the recruitment period. Only after all children had been enrolled was the sequence revealed and the assigned treatment order implemented for each child. This procedure achieves delayed allocation with allocation concealment, while children remained blinded to the assigned order (Single-blind design). The consort flow diagram and the study plan are shown in Supplementary Figures S1 and S2.

At each vision testing visit, the child completed the lens evaluation questionnaire related to his/her subjective visual quality and visual symptoms. A modified version of the Quality of Vision questionnaire^18^ was used to evaluate the specific visual symptoms and complaints. Simulated photographs with descriptive wording were provided to the subjects for a better understanding of the visual symptoms and signs (Supplementary Figure S3).

Part 1 of the study was a prospective crossover study conducted using spectacle lenses mounted in a trial frame. Visual assessments included primary gaze distance and near VA, mid-peripheral near VA, distance and near heterophoria, stereopsis, accommodation and lag of accommodation. Part 2 evaluated mid-peripheral distance VA under different luminance levels with and without glare conditions. As participants were children aged six to 13 years with limited attention spans, completing all assessments in a single session would have required extended testing period and increased the risk of fatigue, which could confound psychophysical measurements and reduce reliability. Therefore mid-peripheral distance VA was scheduled at a separate visit to minimize fatigue. This approach helped to ensure participant comfort and engagement and optimize the validity of the collected data.

### Spectacle Lenses

The SV spectacle lens was made from resin material with 1.60 refractive index. DIMS, and DIMS TED were made from 1.59 polycarbonate material. DIMS is commercially marketed as MiYOSMART spectacle lens by HOYA, Japan. There is a central clear optical zone with refractive power of the wearer (diameter of 9.4 mm). The mid-peripheral zone (diameter of 33 mm) is a honeycomb design zone with + 3.5 D defocus power. The diameter of defocus segment is 1.03 mm with 1.50 mm intervals between each segment.^13^^–^^14^

**Figure 1.**
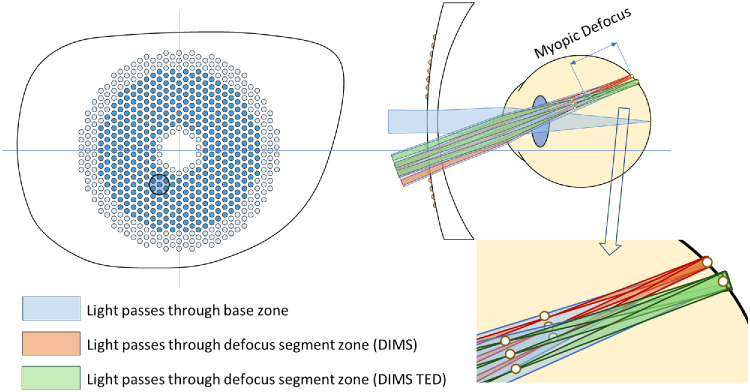
Basic structure and design of DIMS and DIMS TED spectacle lenses. The multiple segments pattern in blue represents the DIMS spectacle lens design while the multiple segments in *blue* and *light blue* represents the DIMS TED spectacle lens design. The treatment zone is enlarged and the central optical zone is reduced in the DIMS TED spectacle lens design while the defocus power is increased from +3.5 D to +4.5 D. *Blue rays* represent the light traces from the central zone of the spectacle lens. *Red rays* represent the light traces from multiple segments of DIMS spectacle lens whereas *green rays* represent light traces from multiple segments of DIMS TED spectacle lens design.

DIMS TED is co-developed by the investigators and Hoya. It has smaller central optical zone (diameter of 6.9 mm). The defocus power of mid-peripheral honeycomb increased from +3.5 D to +4.5 D in DIMS TED and the diameter of the honeycomb design zone increases to approximately 41 mm. The diameter of the defocus segment and the intervals between each segment are the same as in DIMS. Table 1 and Figure 1 show the parameters difference and optical property between DIMS and DIMS TED spectacle lenses. DIMS TED is commercially marketed as MiYOSMART iQ spectacle lens by HOYA, Japan.

**Table 1.** Parameter Differences Between DIMS and DIMS TED Spectacle Lens Design

	DIMS	DIMS TED
Central zone	9.4 mm	6.9 mm
Defocus power	+3.5 D	+4.5 D
Treatment zone defocus segments diameter	33 mm	41 mm
Number of defocus segments	396	630

### Visual Acuity

After dispensing the spectacles, primary gaze distance VA and near VA, as well as mid-peripheral near VA were assessed and repeated after one week of lens wear. VA was measured using Logarithmic 2000 series ETDRS Chart and Low Contrast ETDRS Charts at 4 m (Precision Vision, Woodstock, IL, USA), and Logarithmic Near Visual Acuity Chart 2000 and Logarithmic Near Low Contrast Acuity Chart 2000 at 40 cm, respectively. Because repeated ETDRS visual acuity measurement may have introduced a learning effect, to minimize this, three different ETDRS charts were used, and lens wear order was included as a fixed factor in the Linear Mix Model analysis to control for any time-dependent learning effect.

Both distance VA and near VA were examined under room lighting (500 lux ± 10%) and dim lighting (50 lux ± 10%) illuminance, using high- (100%) and low-contrast (10%) charts. Children were asked to read to the smallest row that they could read. The testing was stopped when only two or fewer letters out of the five letters per row were read correctly. VA was recorded in letter-by-letter logarithm of the minimum angle of resolution (logMAR) notation, each correct letter in the chart representing −0.02 logMAR.

Near VA through the mid-peripheral zone of the spectacle lens under these two levels of illuminance was also measured using high contrast (100%) near visual acuity chart. To ensure the subject was looking through the mid-peripheral zone of the spectacle lens, subject's head was fixed by a chin rest while the Near Visual Acuity Chart was placed superiorly, temporally, inferiorly and nasally away from the visual axis 20°. The set up was the same as our previous study.^19^ These procedures were repeated after wearing the spectacles for one week.

Mid-peripheral distance VA was evaluated separately during a second visit (Part 2 of the study) and was not included in the pre- and post-wear assessments. VA measurements were obtained under room and dim illumination, with and without glare conditions. The glare sources were placed in front of the subjects at 4 m between the ETDRS VA chart (Supplementary Fig. S4). At this final visit (Supplementary Fig. S2), participants were tested with DIMS, DIMS-TED, and SV lenses in a randomised order. The same VA measurement criteria were applied to the mid-peripheral VA.

### Binocularity and Accommodation

Distance and near phoria were measured at 3 m and 33 cm using the Howell phoria distance and near cards. The magnitude (to the nearest 0.5 prism diopter) of Esophoria and Exophoria were recorded as positive and negative values, respectively. For accommodation, only the right eye was assessed. Monocular accommodation was assessed by the push-up method using a Royal Air Force ruler. The examiner gradually advanced the chart toward the subject, who was directed to maintain clear focus on the text and indicate the onset of blur. The mean of three measurements, expressed in diopters, was used for analysis. Accommodative responses were measured with an open-field autorefractor (Shin-Nippon NVision-K5001; Ajinomoto Trading Inc., Tokyo, Japan), which has a refractive resolution of 0.12 D (selectable to 0.25 D) for sphere and cylinder. Children viewed binocularly a printed size of 20/30 letter target at 33 cm, accommodative lag was calculated as the difference between the measured accommodative response and the actual accommodative demand (3D). Stereopsis (in seconds of arc) was evaluated at 40 cm using the Randot Stereotest with Polaroid glasses, with testing halted after the initial error in a series.

### Visual Complaints

Subjects were required to complete the questionnaire about the visual performance after wearing each pair of spectacles for one week. They needed to score the frequency (0 = Never; 1 = Seldom; 2 = Sometimes; 3 = Often; 4 = Always) and the intensity (0 = Not at all; 1 = Mild; 2 = Moderate; 3 = Severe; 4 = Very Severe) of listed symptoms, if any.

Spectacle lenses acceptability was assessed by evaluating the ease of adaptation and the wearer's willingness to use the spectacles after being informed of their potential to slow down myopia progression. (0 = Very satisfied/Strongly agree; 1 = Satisfied/Agree; 2 = Neutral; 3 = Poor/Disagree; 4 = Very poor/Strongly disagree).

### Statistical Analysis

Only data from the right eye were used for the analysis. Given the three-period crossover trial design, all dependent variables were sequentially analyzed in three distinct phases.

Potential carryover effects were evaluated to verify the adequacy washout period between study visits. This was achieved by fitting a linear mixed model (LMM) to the pre-wear baseline measurements of visits two and three, using “Previous Lens Worn” and “Wear Order” as fixed factors, and Subject ID as a random intercept.

To evaluate within-lens temporal changes (the Time effect), the change in VA and binocular functions (post-wear minus pre-wear) was calculated to compare pre-wear baseline and post-wear measurements for each lens type. These change scores were then evaluated using independent, intercept-only LMMs—stratified by Lens Type—to determine whether the temporal shift induced by each specific lens significantly differed from zero.

To conduct the primary efficacy comparisons between three spectacle lenses, an LMM with analysis of covariance approach was used. In these models, the post-wear measurement was designated as the dependent variable, with the corresponding pre-wear measurement included as a continuous baseline covariate. The models incorporated Lens Type (DIMS TED, DIMS and SV) and Wear Order (to control for potential period, learning, or fatigue effects) as fixed factors, and Subject ID as a random intercept. For any variable exhibiting a statistically significant carryover effect during preliminary testing, the primary LMM was mathematically adjusted by incorporating “Previous Lens Worn” as an additional fixed factor to isolate the true treatment effect. When a significant main effect of Lens Type was detected, post-hoc pairwise comparisons were conducted using estimated marginal means with Bonferroni correction.

To accurately quantify the clinical magnitude of significant differences, standardized effect sizes (Cohen's *d*) were calculated by dividing the unstandardized model-estimated mean difference by the pooled raw standard deviation of the specific lens groups being compared. A *P*-value < 0.05 was considered statistically significant for all analyses. All statistical analyses were performed using commercially available software SPSS v.16.0 (SPSS Inc, Chicago, IL, USA).

### Sample Size

Sample size was calculated using G*Power as a conservative approximation for linear mix model. A medium effect size (*f* = 0.25), based on Cohen's conventions for repeated-measures analysis of variance, was assumed to detect clinically meaningful differences between the lenses conditions. The significance Alpha level was set at 0.05, and the statistical power at 90%. The correlation among repeated measures and the non-sphericity correction ε were both set at 0.75, yielding a required sample size of 23 participants. Accounting for a 20% dropout rate, a total of 35 participants were required for this study.

## Results

### Visual Acuity

**Figure 2.**
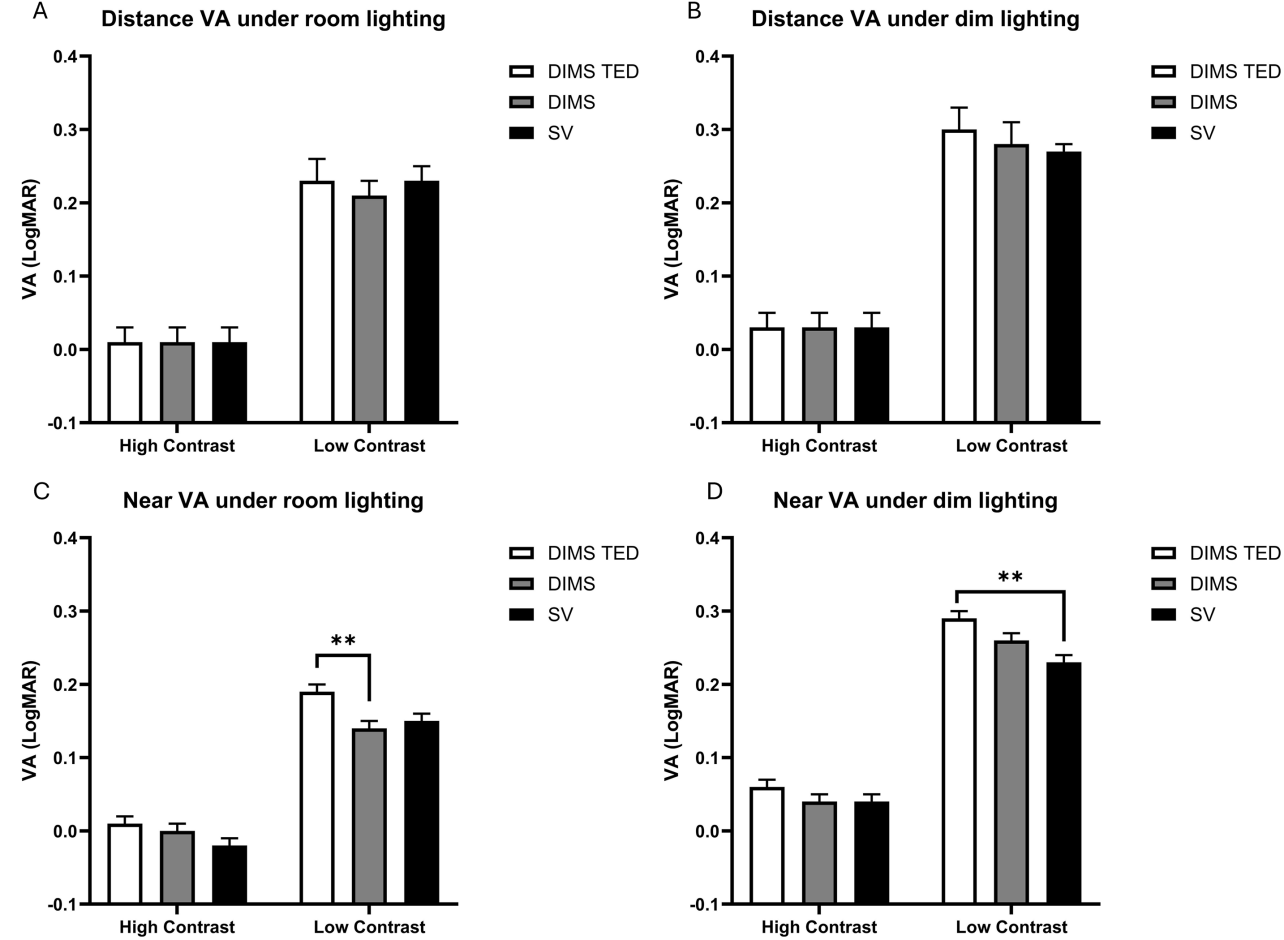
Post-hoc comparison of the central distance and near VA (mean ± standard error) between DIMS, DIMS TED and SV spectacle lenses under room lighting (500 lux ± 10%) and dim lighting (50 lux ± 10%). VA was expressed in logarithm of minimal angle of resolution (logMAR). *P < 0.05; **P < 0.01.

After dispensing the spectacles and wearing the lenses for one week, no statistically significant difference was observed in the primary gaze distance and near VA and mid-peripheral near VA under most testing conditions. There were statistically significant differences in some conditions but the small numerical differences (less than 0.04 logMAR) may not be clinically relevant (Table 2). Furthermore, there were no differences in central distance VA among the three types of spectacle lenses in both high- and low-contrast conditions for both room and dim lighting (*P* > 0.05) (Fig. 2). Central high-contrast near VA was not affected by DIMS TED and DIMS spectacle lenses compared to SV spectacle lenses under both room and dim lighting condition.

**Table 2.** Comparison of the Central Distance and Near VA and Binocular Functions Under Room Lighting (500 lux ± 10%) and Dim Lighting (50 lux ± 10%)

	DIMS TED		DIMS		SV		LMM		Significant Pairwise Differences (Cohen's *d*)	
	Post-Wear EMM [95% CI]	Mean Change [95% CI]	Post-Wear EMM [95% CI]	Mean Change [95% CI]	Post-Wear EMM [95% CI]	Mean Change [95% CI]	Lens Effect	Carryover Effect	DIMS TED Vs. DIMS	DIMS TED Vs. SV
High-Contrast Distance VA										
500 lux	0.01 [−0.02 to 0.05]	−0.01 [−0.04 to 0.02]	0.01 [−0.03 to 0.04]	−0.01 [−0.03 to 0.01]	0.01 [−0.02 to 0.05]	0.01 [−0.01 to 0.03]	0.48	0.31	—	—
50 lux	0.03 [−0.01 to 0.06]	−0.02 [−0.04 to 0.01]	0.03 [0.00–0.06]	0.00 [−0.03 to 0.02]	0.03 [−0.01 to 0.06]	0.01 [−0.01 to 0.02]	0.70	0.15	—	—
Low-contrast distance VA										
500 lux	0.23 [0.18–0.29]	−0.02 [−0.06 to 0.02]	0.21 [0.16–0.26]	−0.03 [−0.07 to 0.00]^†^	0.23 [0.18–0.27]	0.04 [0.01–0.06]^†^	0.72	0.91	—	—
50 lux	0.30 [0.24–0.36]	−0.02 [−0.05 to 0.01]	0.28 [0.23–0.33]	−0.02 [−0.05 to 0.02]	0.27 [0.23–0.32]	0.03 [0.00–0.05]^†^	0.73	0.65	—	—
High-contrast near VA										
500 lux	0.01 [0.00–0.03]	0.01 [−0.01 to 0.02]	0.00 [−0.02 to 0.01]	−0.01 [−0.03 to 0.01]	−0.01 [−0.02 to 0.01]	−0.01 [−0.03 to 0.01]	0.22	0.74	—	—
50 lux	0.06 [0.04–0.08]	−0.01 [−0.03 to 0.02]	0.04 [0.02–0.06]	−0.02 [−0.05 to 0.00]^†^	0.04 [0.03–0.06]	−0.01 [−0.03 to 0.02]	0.21	0.83	—	—
Low-contrast near VA										
500 lux	0.19 [0.16–0.21]	0.02 [−0.02 to 0.05]	0.14 [0.12–0.17]	−0.04 [−0.07 to −0.01]^†^	0.15 [0.12–0.18]	0.00 [−0.02 to 0.02]	<0.01^**^	0.98	0.71	—
50 lux	0.29 [0.26–0.31]	0.00 [−0.03 to 0.03]	0.26 [0.23–0.28]	−0.02 [−0.05 to 0.01]	0.23 [0.21–0.26]	−0.01 [−0.03 to 0.02]	<0.01^**^	0.68	—	0.80
Binocular and accommodation functions										
Distance phoria (∆)	−2.84 [−3.87 to −1.80]	−0.53 [−0.84 to −0.21]^†^	−2.09 [−2.98 to −1.21]	0.16 [−0.20 to 0.52]	−2.99 [−4.06 to −1.92]	−0.47 [−0.86 to −0.09]^†^	0.70	0.66	—	—
Near phoria (∆)	−6.15 [−8.47 to −3.83]	−0.99 [−1.81 to −0.16]^†^	−5.85 [−7.91 to −3.79]	0.03 [−0.75 to 0.80]	−7.15 [−9.59 to −4.71]	−1.00 [−1.69 to −0.31]^†^	0.89	0.17	—	—
Stereopsis (Sec of arc”)	27.19 [24.91–29.47]	−1.71 [−4.47 to 1.05]	26.11 [23.81–28.41]	−1.45 [−3.50 to 0.61]	23.79 [21.48–26.10]	0.79 [−1.49 to 3.07]	0.10	0.20	—	—
Accommodation (D)	13.85 [12.91–14.79]	0.27 [−0.51 to 1.05]	14.36 [13.08–15.63]	−0.33 [−1.09 to 0.43]	14.18 [13.05–15.30]	−0.09 [−0.87 to 0.69]	0.98	0.07	—	—
Lag of accommodation (D)	1.34 [1.15–1.52]	0.01 [−0.18 to 0.20]	1.35 [1.20–1.51]	0.06 [−0.10 to 0.21]	1.47 [1.32–1.63]	0.04 [−0.11 to 0.19]	0.52	0.26	—	—

CI, confidence interval; EMM, estimated marginal mean.

EMM was generated from linear mixed model using analysis of covariance approach, using post-wear measurement as the dependent variable, pre-wear baseline as a continuous covariate, Lens Type and Wear Order as fixed factors, and Subject ID as a random intercept. Mean change [95% CI] represents the estimated shift from pre-wear baseline to post-wear (post-measurement minus pre-measurement). For variables exhibiting a significant main effect of lens type, post-hoc pairwise differences with Bonferroni corrections are reported alongside standardized effect sizes (Cohen's *d*). Effect sizes were calculated by dividing the unstandardized model-estimated mean difference by the pooled raw standard deviation of the specific lens groups being compared.

* *P* < 0.05 from lens type effect.

** *P* < 0.01 from lens type effect.

† Statistically significant change from pre-wear baseline to post-wear (p < 0.05), calculated via independent intercept-only LMMs using individual change scores.

Under room lighting, low-contrast near VA was reduced in DIMS TED spectacle lenses compared to DIMS spectacle lenses by 0.06 ± 0.10 logMAR (*P* < 0.01). Under dim lighting conditions, low-contrast near VA was reduced by 0.06 ± 0.11 logMAR in DIMS TED spectacle lenses compared to SV spectacle lenses (*P* < 0.01) (Table 2).

**Figure 3.**
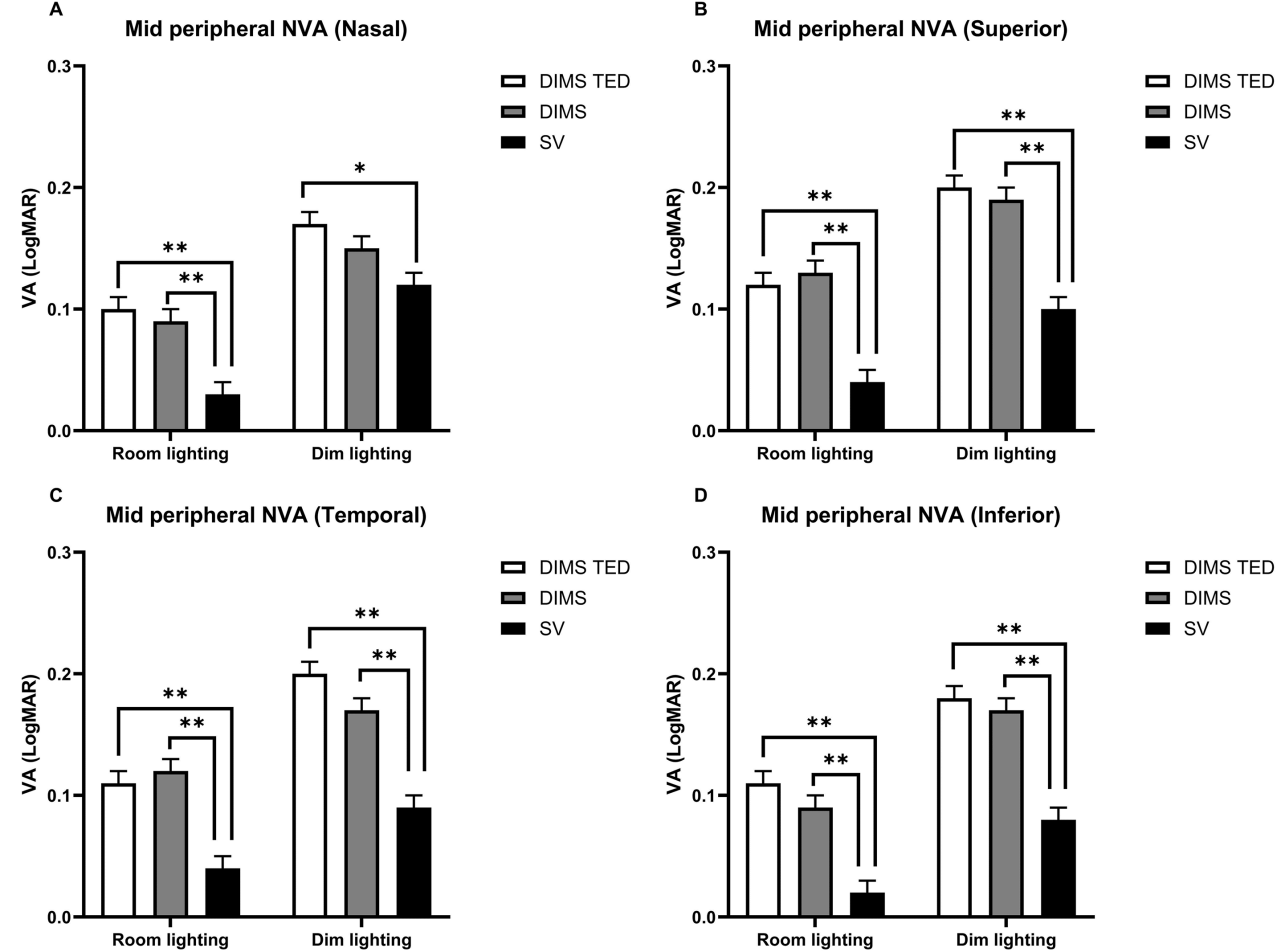
Post-hoc pairwise comparison of the mid-peripheral Near VA (mean ± standard error) between DIMS, DIMS TED and SV spectacle lenses under room lighting (500 lux ± 10%) and dim lighting (50 lux ± 10%) in different quadrants. *P < 0.05; **P < 0.01.

For mid-peripheral near VA, both DIMS TED and DIMS spectacle lenses showed statistically significant reduction compared to SV spectacle lenses across all four quadrants under both lighting conditions with the exception of the nasal quadrant between DIMS and SV spectacle lenses under dim lighting (Fig. 3). There was no difference between DIMS TED and DIMS spectacle lenses in any quadrants.

**Figure 4.**
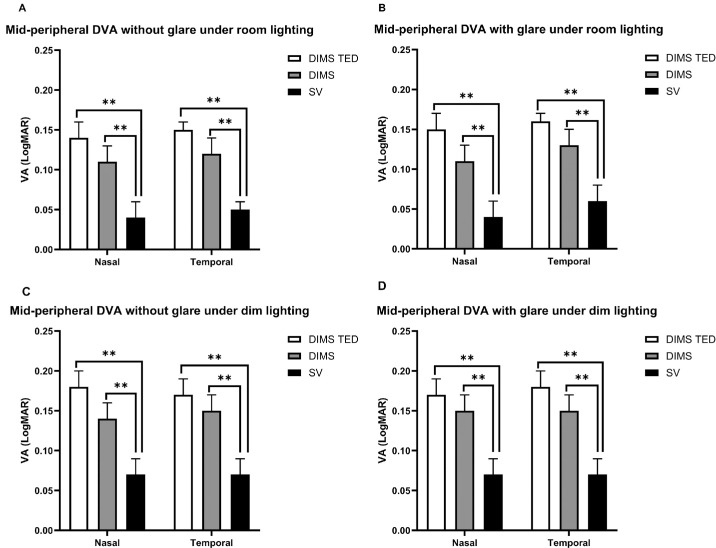
Post-hoc pairwise comparison of the mid-peripheral Distance VA (mean ± standard error) between DIMS, DIMS TED and SV spectacle lenses under room lighting (500 lux ± 10%) and dim lighting (50 lux ± 10%) in nasal and temporal quadrants with and without glare conditions. *P < 0.05; **P < 0.01.

Reduction was observed in mid-peripheral distance VA in both DIMS TED and DIMS spectacle lenses compared to SV spectacle lenses under both room lighting and dim lighting conditions (*P* < 0.01) (Table 3). The reduction in mid-peripheral distance VA for DIMS TED and DIMS spectacle lenses was consistent in both nasal and temporal directions compared to SV spectacle lenses, regardless of the presence or absence of glare (Fig. 4). Similar results were observed under dim lighting conditions. There was no difference in mid-peripheral distance VA between DIMS TED and DIMS spectacle lenses (*P* > 0.05) (Table 4).

**Table 3.** Comparison of the Mid-Peripheral Near VA in Different Positions at the Dispensing Visit (Pre-) and After Wearing the Spectacles One Week (Post-) Under Room Lighting (500 lux ± 10%) and dim lighting (50 lux ± 10%).

	DIMS TED		DIMS		SV		LMM		Significant Pairwise Differences (Cohen's *d*)	
	Post-Wear EMM [95% CI]	Mean Change [95% CI]	Post-Wear EMM [95% CI]	Mean Change [95%CI]	Post-Wear EMM [95% CI]	Mean Change [95% CI]	Lens Effect	Carry-Over Effect	DIMS TED Vs. SV	DIMS Vs. SV
Nasal										
500 lux	0.10 [0.07–0.12]	0.00 [−0.03 to 0.02]	0.09 [0.06–0.12]	−0.02 [−0.04 to 0.01]^†^	0.03 [0.01–0.05]	−0.02 [−0.04 to 0.00]	<0.01^**^	0.26	1.06	0.86
50 lux	0.17 [0.15–0.19]	0.00 [−0.02 to 0.02]	0.15 [0.13–0.18]	−0.02 [−0.05 to 0.00]	0.12 [0.11–0.14]	−0.01 [−0.03 to 0.01]	<0.01^**^	0.03^‡^	0.71	
Superior										
500 lux	0.12 [0.10–0.14]	−0.01 [−0.02 to 0.01]	0.13 [0.10–0.16]	−0.01 [−0.04 to 0.01]	0.04 [0.02–0.06]	−0.01 [−0.03 to 0.01]	<0.01^**^	0.40	1.34	1.29
50 lux	0.20 [0.18–0.21]	−0.01 [−0.03 to 0.01]	0.19 [0.17–0.22]	−0.01 [−0.03 to 0.02]	0.10 [0.08–0.12]	0.00 [−0.02 to 0.02]	<0.01^**^	<0.05^‡^	1.50	1.28
Temporal										
500 lux	0.11 [0.09–0.13]	−0.01 [−0.03 to 0.00]	0.12 [0.10–0.14]	−0.01 [−0.03 to 0.02]	0.04 [0.02–0.06]	−0.02 [−0.03 to 0.00]	<0.01^**^	0.17	1.28	1.36
50 lux	0.20 [0.18–0.23]	0.00 [−0.02 to 0.01]	0.17 [0.15–0.19]	−0.02 [−0.05 to 0.00]^†^	0.09 [0.07–0.12]	−0.01 [−0.02 to 0.01]	<0.01^**^	0.13	1.62	1.20
Inferior										
500 lux	0.11 [0.09–0.13]	0.00 [−0.02 to 0.02]	0.09 [0.07–0.12]	−0.02 [−0.05 to 0.00]	0.02 [0.00–0.05]	−0.04 [−0.06 to −0.01]^†^	<0.01^*^	0.56	1.45	1.07
50 lux	0.18 [0.16–0.20]	−0.01 [−0.03 to 0.01]	0.17 [0.15–0.20]	−0.02 [−0.05 to 0.01]^†^	0.08 [0.06–0.10]	−0.02 [−0.04 to 0.00]^†^	<0.01^*^	0.04^‡^	1.61	0.99

EMM was generated from linear mixed model using analysis of covariance approach, using post-wear measurement as the dependent variable, pre-wear baseline as a continuous covariate, Lens Type and Wear Order as fixed factors, and Subject ID as a random intercept. Mean change [95% CI] represents the estimated shift from pre-wear baseline to post-wear (post-measurement minus pre-measurement). For variables exhibiting a significant main effect of lens type, post-hoc pairwise differences with Bonferroni corrections are reported alongside standardized effect sizes (Cohen's *d*). Effect sizes were calculated by dividing the unstandardized model-estimated mean difference by the pooled raw standard deviation of the specific lens groups being compared.

* *P* < 0.05 from lens type effect.

** *P* < 0.01 from lens type effect.

† Statistically significant change from pre-wear baseline to post-wear (*P* < 0.05), calculated via independent intercept-only LMMs using individual change scores.

‡ Statistically significant carryover effect (*P* < 0.05) from the previously worn lens, determined before primary analysis. For these specific variables, the primary LMM and resulting main lens effect *P*-value were mathematically adjusted by incorporating “Previous Lens Worn” as an additional fixed factor.

**Table 4.** Comparison of the Mid-Peripheral Distance VA at the Nasal and Temporal Positions Under Room Lighting (500 lux ± 10%) and Dim Lighting (50 lux ± 10%) With and Without Glare Conditions

					Significant Pairwise Differences (Cohen's *d*)	
	DIMS TED, Mean [95% CI]	DIMS, Mean [95% CI]	SV, Mean [95% CI]	LMM Lens Effect	DIMS TED Vs. SV	DIMS Vs. SV
500 lux						
Nasal						
Without glare	0.14 [0.10–0.17]	0.11 [0.07–0.14]	0.05 [0.02–0.09]	<0.01^**^	0.86	0.54
With glare	0.15 [0.11–0.18]	0.11 [0.08–0.14]	0.06 [0.03–0.09]	<0.01^**^	0.87	0.49
Temporal						
Without glare	0.15 [0.12–0.17]	0.13 [0.10–0.15]	0.06 [0.03–0.09]	<0.01^**^	1.02	0.79
With glare	0.15 [0.12–0.19]	0.13 [0.10–0.16]	0.07 [0.04–0.10]	<0.01^**^	0.99	0.65
50 lux						
Nasal						
Without glare	0.17 [0.14–0.21]	0.14 [0.11–0.18]	0.08 [0.05–0.12]	<0.01^**^	0.86	0.62
With glare	0.17 [0.13–0.21]	0.15 [0.11–0.18]	0.09 [0.05–0.12]	<0.01^**^	0.84	0.58
Temporal						
Without glare	0.17 [0.14–0.20]	0.15 [0.11–0.18]	0.09 [0.05–0.12]	<0.01^**^	0.84	0.73
With glare	0.17 [0.14–0.21]	0.16 [0.12–0.19]	0.08 [0.04–0.11]	<0.01^**^	0.95	0.81

* *P* < 0.05 from lens type effect.

** *P* < 0.01 from lens type effect.

### Binocular and Accommodation Functions

No differences were observed in the binocular function tests (distance and near phoria, stereopsis, accommodation and lag of accommodation) between pre- and post-wearing of the spectacles after one week with the exception of distance and near phoria in SV and DIMS TED spectacle lenses (Table 2). After wearing the spectacles for one week, there was no difference in binocular functions between the three types of spectacle lenses (Table 2).

### Visual Complaints

**Figure 5.**
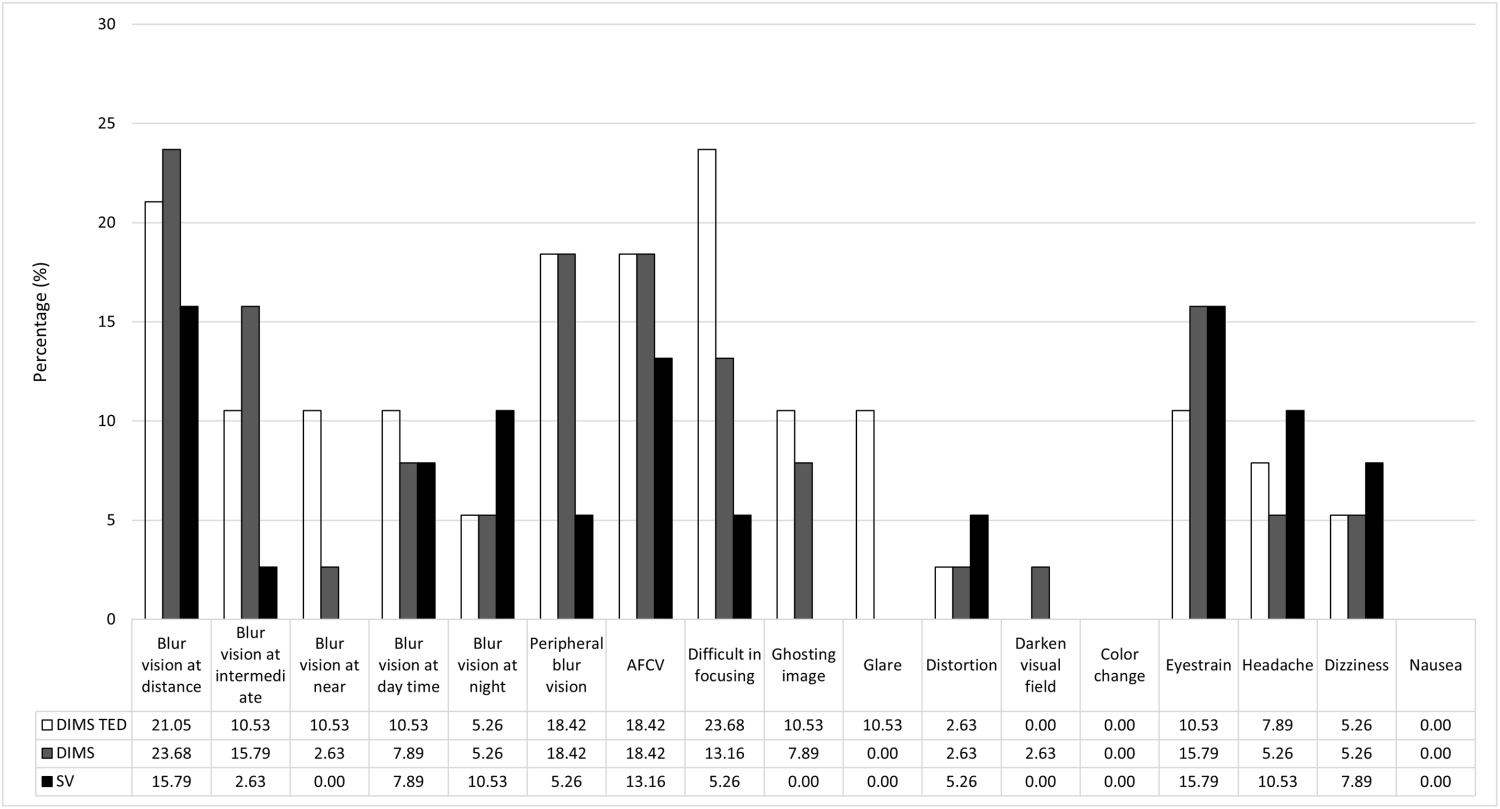
Subjective response to the frequency of visual complaints (from sometimes to always). AFCV, adjust frame to get clear vision.

**Figure 6.**
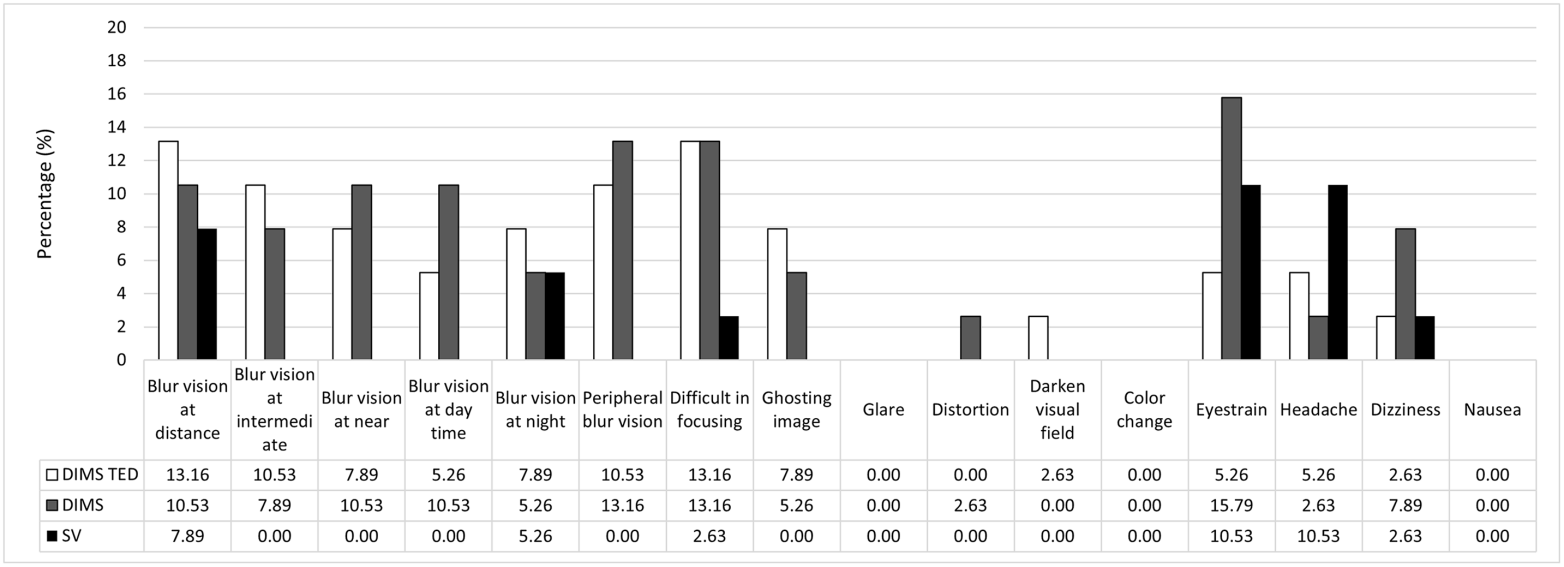
Subjective response to the severity of visual complaints (from moderate to very severe).

**Figure 7.**
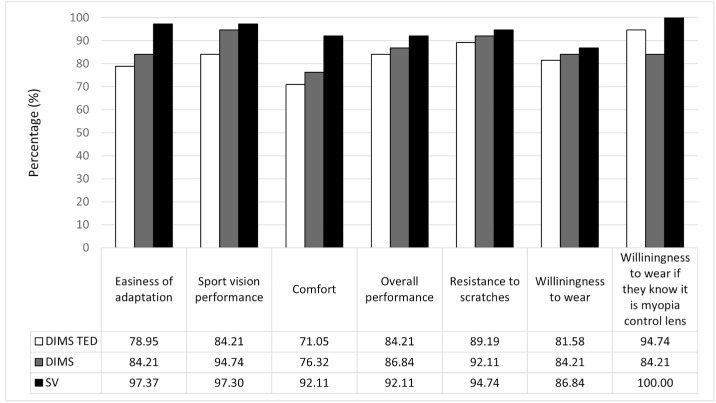
Rating of spectacle lenses performance after wearing different types of spectacle lenses for one week. The percentage represents the ratings from subjects (from very satisfied to satisfied or strongly agree to agree).

Figure 5 shows the scores of how frequent the subjects experienced the visual symptoms (sometimes to always inclusive) after wearing the spectacles for one week. Subjects wearing the two test spectacle lenses (DIMS and DIMS TED) reported greater difficulty with focusing, increased peripheral blur, blurred vision at intermediate and near distances, and ghosting images compared with those wearing the SV spectacle lenses. Figure 6 shows the scores of how severe of the visual symptoms experienced by the children (moderate to very severe inclusive). Difficult with focusing, peripheral blur vision, blur vision at intermediate and near as well as ghosting images were more often in DIMS and DIMS TED spectacle lenses compared to SV. However, the severity scores were similar among the three types of spectacle lenses. The overall ratings of three types of spectacle lenses, including the easiness of adaptation, comfort and willingness to wear were similar among three spectacle lenses (Fig. 7). Interestingly, there was an increase in willingness to wear the DIMS TED spectacle lenses if the subject know that the particular spectacles were for myopia control. This may imply subjects can endure the mild visual disturbance from DIMS TED spectacle lenses.

## Discussion

The present study evaluated the visual performance of the newly developed DIMS TED spectacle lens compared with DIMS and SV lenses. It is important to note that this crossover study did not assess myopia control efficacy or long-term adaptation, but rather aimed to evaluate the short-term visual performance to better understand the balance between potential visual compromises and the anticipated benefits of myopia control.

### Visual Acuity Comparisons

Central distance and near VA as well as mid-peripheral near VA were measured at the spectacles dispensing visit and after wearing the spectacles for one week. No statistical or clinically significant improvement was found in the majority conditions between the three types of the spectacle lenses after one-week wear. Although there were statistically significant differences between pre- and post- VA in some different lens types, the mean differences were just about 2 to 3 letters which could be considered as clinically insignificant (Table 2). This is also confirmed with another short-term adaptation study by Lu et al.^20^ They found the central distance vision did not show significant changes after wearing the DIMS and SV spectacle lenses one week. Lam et al.^21^ reported a statistically significant improvement (−0.09 ± 0.06 LogMAR) in best corrected high contrast distance VA for both DIMS and SV spectacle lenses wearer for long-term adaptation of 2 years. It has been reported adaptation of DIMS spectacles in young children took about a week,^21^ the longer they wear the spectacles, the better they adapted to the lenses. The findings from one week of lens wear indicated the DIMS TED lens is promising for adaptation in young children. Further improvement may also occur with longer periods of spectacle wear.

After one week of lens wear, no differences were observed among three types of spectacle lenses in central high- or low-contrast distance VA or in central high-contrast near VA, under either room or dim illumination conditions (*P* > 0.05, Table 2). Previous studies have similarly reported that DIMS spectacle lenses provided comparable primary gaze VA to single vision spectacle lenses.^14^^,^^20^^–^^23^ suggesting that the central clear optical zone does not adversely affect VA.

DIMS TED is based on the Defocus Incorporated Multiple Segments Technology used in the DIMS spectacle lens (commercially marked as MiYOSMART), but incorporates a higher myopic defocus power of +4.5 D. The central optical zone of DIMS TED is reduced from 9.4 mm in DIMS to 6.9 mm. Despite the smaller central zone, primary gaze VA is expected to remain well preserved, provided the optical centre is properly alighted with the visual axis. However, children with larger pupil size may be exposed to the defocus segments even in primary gaze.^24^^–^^25^ Furthermore, the reduced central clear optical zone may lead to poorer primary gaze VA if lens fitting is suboptimal, such as when the spectacles slip downward.

Under standard illumination, DIMS TED spectacle lenses showed a reduction in central low-contrast near VA compared with DIMS lenses (Fig. 2), while under dim lighting, there was reduction in central low-contrast near VA compared to SV spectacle lenses (Fig. 2). And no difference was found between DIMS TED and DIMS spectacle lenses. This may be attributed to increased pupil size under dim lighting, allowing greater exposure to the multiple segments’ zones, increasing the influence of higher-order aberrations on image quality. As a result, a similar reduction in low-contrast central near VA was observed between DIMS TED and DIMS spectacle lenses.^26^ In summary, although statistically significant differences were observed, a change of around 0.05–0.10 logMAR may not represent clinically meaningful visual impairment.

Unsurprisingly, both DIMS and DIMS TED spectacle lenses showed slightly poorer mid-peripheral distance and near VA (about a line worse) than SV spectacle lenses under each illumination level (Figs. 3, 4). Li et al.^27^ reported that the peripheral near VA was reduced by 0.06 to 0.07 logMAR in highly aspheric lenslets (HALT) while it was reduced by 0.09 logMAR in DIMS lenses compared to SV lenses. Our previous study^19^ found that the peripheral near VA was decreased by 0.05 logMAR in DIMS lenses compared to SV lenses when asking subject to move their eyes toward the lenslets zone in DIMS lenses. These findings are consistent with the current results, in which the mid-peripheral distance and near VA in DIMS TED spectacle lenses were reduced approximately 0.10 logMAR compared to SV spectacle lenses. Although the mid-peripheral distance and near VA was about a line worse in DIMS TED and DIMS spectacle lenses than the SV spectacle lenses, the reduction may not be clinically meaningful. Rosser et al.^28^ reported that a 0.10 logMAR change could not be distinguished from test-retest variability with high sensitivity while the smallest reliably detectable and clinically meaningful difference is estimated as a change of 0.20 logMAR using ETDRS visual acuity chart. Noushad et al.^29^ suggested that the 95% confidence limit for test-retest variability using standard logMAR VA chart was approximately ±0.08 to ±0.10 logMAR. Therefore, although statistically significant reduction in mid-peripheral distance and near VA were observed with DIMS TED and DIMS spectacle lenses compared to SV spectacle lenses, the magnitude of change may not represent a clinically meaningful visual acuity drop. These findings may suggest the increase defocus power in DIMS TED lenses does not compromise peripheral vision performance.

Fernandes et al.^30^ demonstrated that multifocal optics induce a short-term delay in neural response, a phenomenon that likely originates at the retinal level, where photoreceptor and bipolar cell layers are required to process superimposed signals characterized by differing spatial coherence. The split of incoming light into a clear central vision zone and scattered defocus zone may cause localized delays in neural signaling from the retina and may explain the temporary slight decrease in visual performance by DIMS and DIMS TED lenses.

Several studies have reported similar reduction of mid-peripheral VA across different retinal quadrants in both adults and children and some showed even a higher drop in VA.^20^^,^^22^^,^^31^ Despite the higher defocus power, DIMS TED lenses showed only slight drop in mid-peripheral distance and near VA compared to DIMS spectacle lenses, equivalent to about one to two letters.

Previous studies found that children generally adapt better to DIMS spectacle lenses than adults. One possible explanation is the larger pupil size in children,^24^^,^^32^ which may reduce overlap between the central clear zones and the defocus segments zones during eye movement. In real life situation, children are also unlikely to view constantly through the defocus zone when they need to perform visual demanding tasks or reading. Kaymak et al.^22^ suggested that such decrease in mid-peripheral VA is similar to the intermediate zone of the progressive lenses which are reportedly safe and tolerable.^33^^,^^34^ This is further affirmed from the findings in the visual symptom's questionnaire.

Children wearing myopia control spectacles have been reported to adapt to changes in the visual quality within days to weeks.^35^ Blur Adaptation Theory suggests a short period of exposure to myopic defocus can improve VA through neural compensatory mechanisms.^36^^,^^37^ Kang et al.^36^ found measurable improvement in VA within four minutes of exposure to defocus. A previous study which investigated children's initial impressions of cylindrical annular refractive elements (CARE) lenses indicated that CARE lenses are less favorable than those of SV lenses at one week. However, by three months, no significant differences between the two lens types were observed except for near vision.^38^ Ghosh et al.^39^ found myopes exhibited a greater parafoveal blur adaptation than emmetropes. Long-term adaptation has also been observed in DIMS lens wearers over two years.^21^ Therefore further studies are needed to determine the extent and time course of any neural adaptation.

### Binocular and Accommodation Functions

The results indicated that there was no significant difference in most of the binocular functions between three types of the spectacle lenses after wearing each type of the spectacle lenses for one week. Although there was a mild shift in distance and near exophoria between pre- and post-wearing of the DIMS TED and SV spectacle lenses, the magnitude was less than 1 prism diopters (Table 2). Earlier study by Lam et al.^21^ reported no significant difference in distance and near phoria in children wearing DIMS spectacle lenses over two years, suggesting the mild shift in exophoria may not be clinically significant when subject wear the spectacle lenses for longer term. Monocular and binocular accommodation and accommodative lag were reduced over two years and suggested it may be due to the age effect as such effect was also noticed in children wearing SV spectacle lenses. Reduction in accommodative lag was also noted but found not related to the treatment group (DIMS spectacle lenses). In the current study, there was no difference in amplitude of accommodation and accommodation lag between three types of spectacle lenses. It may imply that the DIMS and DIMS TED spectacle lenses did not affect the wearer's accommodative status. There was no difference in stereopsis between three types of spectacle lenses. In the earlier study^21^ a slight improvement in stereopsis for both DIMS and SV spectacle lenses group over two years was noted, although it was not clinically significant (5.9 sec of arc and 7.4 sec of arc). Perhaps DIMS TED spectacle lenses may have similar trend in improvement of stereopsis with time.

### Acceptance of DIMS TED Spectacle Lenses

The frequency and intensity of visual complaints were similar between three types of spectacle lenses in this study (Figs. 5, 6). Some differences between DIMS TED and SV spectacle lenses were noted for examples, in experiencing blur vision in intermediate distance (10.5% vs. 2.6%), blur vision at near (10.53% vs. 0%), difficult in focusing (23.7% vs. 5.3%) and peripheral blur vision (18.4% vs. 5.3%) (Fig. 5). Among these visual complaints, subjects with DIMS TED spectacle lenses reported the severity from moderate to very severe while subjects with SV spectacle lenses reported mild intensity (Fig. 6). It is predictable subjects with DIMS TED spectacle lenses experienced these visual complaints because of the increased defocus power and small central optical zone. A majority of the children were willing to wear three types of spectacle lenses if they know these lenses are designed for myopia control. More than 90% of the children were willing to wear DIMS TED and SV spectacle lenses while more than 80% of the children would like to wear DIMS spectacle lenses. Almost all children thought it was easy to adapt the single-vision spectacle lenses while around 80% children also agreed the DIMS and DIMS TED spectacle lenses were easy to adapt (Fig. 7). All test lenses were well tolerated and accepted by the children generally in the current study. These findings suggested a reasonable representation of visual performance in the real-world situations.

### Limitations

The study has several limitations. First, no formal washout period was included between lenses wear periods. In traditional crossover designs, a washout period is used to eliminate the risk of carryover effects from the preceding intervention.^40^^,^^41^ To address this, we statistically evaluated the baseline (pre-wear) measurements across periods and found no significant differences based on the previously worn lens, suggesting no detectable carryover effect on our specific visual and binocular outcomes. Future studies should incorporate a standard washout period to definitively isolate the effects of each lens design. Second, potential industry bias may exist due to commercial funding, although data oversight and analyses were conducted independently by the academic investigators. Third, the study did not assess real-world visual performance under dynamic conditions and visual challenges such as fluctuating illumination, glare, and moving targets and small sample size may not represent the broader population. Future studies should incorporate functional testing, such as mobility mazes or virtual reality simulations.

Finally, this short-term study was designed to evaluate the feasibility and validity of proceeding to a future randomized clinical trial for DIMS TED spectacle lenses. The long-term effects on visual adaptation and myopia control efficacy remain to be investigated.

## Conclusions

Central vision with DIMS TED and DIMS spectacle lenses were comparable to that of standard single vision lenses, although the multiple segments design may reduce mid-peripheral distance and near VA. Most participants reported good acceptance and willingness to wear the test lenses if they were effective in controlling myopia progression. No adverse effects on the measured visual functions were observed. The visual performance of the DIMS TED spectacle lenses appears promising and warrants further investigation to evaluate its efficacy in myopia control and long-term impact on visual functions and myopia management.

## Supplementary Material

Supplement 1

Supplement 2

Supplement 3

Supplement 4

## References

[1] Fricke TR, Jong M, Naidoo KS, et al. Global prevalence of visual impairment associated with myopic macular degeneration and temporal trends from 2000 through 2050: systematic review, meta-analysis and modelling. *Br J Ophthalmol*. 2018; 102: 855–862. 29699985 10.1136/bjophthalmol-2017-311266 PMC6047154

[2] Holden BA, Fricke TR, Wilson DA, et al. Global prevalence of myopia and high myopia and temporal trends from 2000 through 2050. *Ophthalmology*. 2016; 123: 1036–1042. 26875007 10.1016/j.ophtha.2016.01.006

[3] Wallman J, Winawer J. Homeostasis of eye growth and the question of myopia. *Neuron**.* 2004; 43: 447–468. 15312645 10.1016/j.neuron.2004.08.008

[4] Liu Y, Wildsoet C. The effect of two-zone concentric bifocal spectacle lenses on refractive error development and eye growth in young chicks. *Invest Ophthalmol Vis Sci**.* 2011; 52: 1078–1086. 20861487 10.1167/iovs.10-5716 PMC3053095

[5] Tse DY, Lam CS, Guggenheim JA, et al. Simultaneous defocus integration during refractive development. *Invest Ophthalmol Vis Sci**.* 2007; 48: 5352–5359. 18055781 10.1167/iovs.07-0383

[6] McFadden SA, Tse DY, Bowrey HE, et al. Integration of defocus by dual power Fresnel lenses inhibits myopia in the mammalian eye. *Invest Ophthalmol Vis Sci**.* 2014; 55: 908–917. 24398103 10.1167/iovs.13-11724 PMC3926275

[7] Arumugam B, Hung LF, To CH, et al. The effects of simultaneous dual focus lenses on refractive development in infant monkeys. *Invest Ophthalmol Vis Sci**.* 2014; 55: 7423–7432. 25324283 10.1167/iovs.14-14250 PMC4575087

[8] Smith EL III, Kee CS, Ramamirtham R, et al. Peripheral vision can influence eye growth and refractive development in infant monkeys. *Invest Ophthalmol Vis Sci*. 2005; 46: 3965–3972. 16249469 10.1167/iovs.05-0445 PMC1762100

[9] Atchison DA, Rosen R. The possible role of peripheral refraction in development of myopia. *Optom Vis Sci**.* 2016; 93: 1042–1044. 27560691 10.1097/OPX.0000000000000979

[10] Yam JC, Zhang XJ, Zhang Y, et al. Three-year clinical trial of low-concentration atropine for myopia progression (LAMP) study: continued versus washout: phase 3 report. *Ophthalmology**.* 2022; 129: 308–321. 34627809 10.1016/j.ophtha.2021.10.002

[11] Cavuoto KM, Trivedi RH, Prakalapakorn SG, et al. Multifocal soft contact lenses for the treatment of myopia progression in children: a report by the American Academy of Ophthalmology. *Ophthalmology**.* 2025; 132: 495–503. 39503665 10.1016/j.ophtha.2024.09.031 PMC11930616

[12] Lawrenson JG, Shah R, Huntjens B, et al. Interventions for myopia control in children: a living systematic review and network meta-analysis. *Cochrane Database Syst Rev**.* 2023; 2(2): CD014758. 36809645 10.1002/14651858.CD014758.pub2 PMC9933422

[13] Lam CSY, Tang WC, Tse DY, et al. Defocus incorporated multiple segments (DIMS) spectacle lenses slow myopia progression: a 2-year randomised clinical trial. *Br J Ophthalmol*. 2020; 104: 363–368. 31142465 10.1136/bjophthalmol-2018-313739 PMC7041503

[14] Lam CSY, Tang WC, Zhang HY, et al. Long-term myopia control effect and safety in children wearing DIMS spectacle lenses for 6 years. *Sci Rep**.* 2023; 13: 5475. 37015996 10.1038/s41598-023-32700-7 PMC10073092

[15] Walline JJ, Walker MK, Mutti DO, et al. Effect of high add power, medium add power, or single-vision contact lenses on myopia progression in children: the BLINK Randomized Clinical Trial. *JAMA**.* 2020; 324: 571–580. 32780139 10.1001/jama.2020.10834 PMC7420158

[16] Zhang H, Leung KY, Leung M, et al. Myopia control using a modified optical defocus soft contact lens in schoolchildren-A 12-month randomised double masked control trial. *Ophthalmic Physiol Opt**.* 2025; 45: 969–981. 40167121 10.1111/opo.13501 PMC12087850

[17] Nallour Raveendran R, Ong WS, Wong YL, et al. Effect of increased power and asphericity of highly aspherical lenslets on myopia control efficacy: a contralateral crossover study. *Transl Vis Sci Technol*. 2025; 14(11): 9. 10.1167/tvst.14.11.9 PMC12614271 41222192

[18] McAlinden C, Pesudovs K, Moore JE. The development of an instrument to measure quality of vision: the Quality of Vision (QoV) questionnaire. *Invest Ophthalmol Vis Sci**.* 2010; 51: 5537–5545. 20505205 10.1167/iovs.10-5341

[19] Liu KKK, Zhang HY, Leung DKY, et al. Evaluation of the peripheral visual performance of DIMS spectacle lenses versus single vision lenses. *Front Neurosci*. 2024; 18: 1460062. 39691627 10.3389/fnins.2024.1460062 PMC11650793

[20] Lu Y, Lin Z, Wen L, et al. The adaptation and acceptance of Defocus Incorporated Multiple Segment lens for Chinese children. *Am J Ophthalmol*. 2020; 211: 207–216. 31837317 10.1016/j.ajo.2019.12.002

[21] Lam CSY, Tang WC, Qi H, et al. Effect of Defocus Incorporated Multiple Segments spectacle lens wear on visual function in myopic Chinese children. *Transl Vis Sci Technol**.* 2020; 9(9): 11. 10.1167/tvst.9.9.11 PMC7442864 32879767

[22] Kaymak H, Neller K, Schütz S, et al. Vision tests on spectacle lenses and contact lenses for optical myopia correction: a pilot study. *BMJ Open Ophthalmol*. 2022; 7: e000971. 10.1136/bmjophth-2022-000971 PMC8984052 35464151

[23] Gao Y, Lim EW, Yang A, et al. The impact of spectacle lenses for myopia control on visual functions. *Ophthalmic Physiol Opt*. 2021; 41: 1320–1331. 34529275 10.1111/opo.12878 PMC9291741

[24] Guillon M, Dumbleton K, Theodoratos P, et al. The effects of age, refractive status, and luminance on pupil size. *Optom Vis Sci**.* 2016; 93: 1093–1100. 27232893 10.1097/OPX.0000000000000893 PMC5006796

[25] Dolce JC, Nti AN, Berntsen DA. The effect of pupil size on visual performance with center-distance soft multifocal contact lenses. *Optom Vis Sci**.* 2025; 102: 421–426 40505043 10.1097/OPX.0000000000002271 PMC13020428

[26] Gregory HR, Nti AN, Wolffsohn JS, et al. Visual performance of center-distance multifocal contact lenses fit using a myopia control paradigm. *Optom Vis Sci**.* 2021; 98: 272–279 33771957 10.1097/OPX.0000000000001665 PMC8007064

[27] Li X, Ding C, Li Y, et al. Influence of lenslet configuration on short-term visual performance in myopia control spectacle lenses. *Front Neurosci*. 2021; 15: 667329. 34113234 10.3389/fnins.2021.667329 PMC8185291

[28] Rosser DA, Cousens SN, Murdoch IE, et al. How sensitive to clinical change are ETDRS logMAR visual acuity measurements? *Invest Ophthalmol Vis Sci**.* 2003; 44: 3278–3281. 12882770 10.1167/iovs.02-1100

[29] Noushad B, Thomas J, Amin SV. Reliability of a modified logMAR distant visual acuity chart for routine clinical use. *Oman J Ophthalmol*. 2012; 5;87. 22993462 10.4103/0974-620X.99370 PMC3441035

[30] Fernandes P, Ferreira C, Domingues J, et al. Short-term delay in neural response with multifocal contact lens might start at the retinal level. *Doc Ophthalmol*. 2022; 145: 327–336 10.1007/s10633-022-09870-2 35364776

[31] Desiato A, Lam HY, Anand RR, et al. The impact of myopia control spectacle lens designs on visual function. *Ophthalmic Physiol Opt**.* 2026; 46: 49–59. 41784766 10.1007/s44402-026-00026-2 PMC13369762

[32] Winn B, Whitaker D, Elliott DB, et al. Factors affecting light-adapted pupil size in normal human subjects. *Invest Ophthalmol Vis Sci**.* 1994; 35: 1132–1137. 8125724

[33] Forkel J, Reiniger JL, Muschielok A, et al. Personalized progressive addition lenses: correlation between performance and design. *Optom Vis Sci**.* 2017; 94: 208–218. 27918396 10.1097/OPX.0000000000001016

[34] Sheedy JE, Campbell C, King-Smith E, et al. Progressive powered lenses: the Minkwitz theorem. *Optom Vis Sci**.* 2005; 82: 916–922. 16276325 10.1097/01.opx.0000181266.60785.c9

[35] Fatimah M, Agarkar S, Narayanan A. Impact of defocus incorporated multiple segments (DIMS) spectacle lenses for myopia control on quality of life of the children: a qualitative study. *BMJ Open Ophthalmol*. 2024; 9(1): e001562. 10.1136/bmjophth-2023-001562 PMC11227816 38960416

[36] Kang MT, Wang B, Ran AR, et al. Brain activation induced by myopic and hyperopic defocus from spectacles. *Front Hum Neurosci.* 2021; 15: 711713. 34594194 10.3389/fnhum.2021.711713 PMC8477670

[37] Khan KA, Dawson K, Mankowska A, et al. The time course of blur adaptation in emmetropes and myopes. *Ophthalmic Physiol Opt**.* 2013; 33: 305–310. 23662962 10.1111/opo.12031

[38] Alvarez-Peregrina C, Sanchez-Tena MA, Villa-Collar C, et al. Clinical evaluation of MyoCare in Europe (CEME) for myopia management: One-year results. *Ophthalmic Physiol Opt**.* 2025; 45: 1025–1035. 40296784 10.1111/opo.13517 PMC12087851

[39] Ghosh A, Zheleznyak L, Barbot A, et al. Neural adaptation to peripheral blur in myopes and emmetropes. *Vis Res**.* 2017; 132: 69–77. 27919674 10.1016/j.visres.2016.09.017 PMC8094114

[40] Sturdevant SG, Lumley T. Statistical methods for testing carryover effects: a mixed effects model approach. *Contemp Clin Trials Commun*. 2021; 22: 100711. 33997456 10.1016/j.conctc.2021.100711 PMC8102872

[41] Lim CY, In J. Considerations for crossover design in clinical study. *Korean J Anesthesiol*. 2021; 74: 293–299. 34344139 10.4097/kja.21165 PMC8342834

