# Supplementary figures and images for: Visual Performance of Defocus Incorporated Multiple Segments With Triple Enhanced Design Spectacle Lenses Versus DIMS and Single Vision Lenses
Source: Transl Vis Sci Technol. 2026 Jul 29;15(7):32. doi: 10.1167/tvst.15.7.32 (PMC13426864; doi:10.1167/tvst.15.7.32)

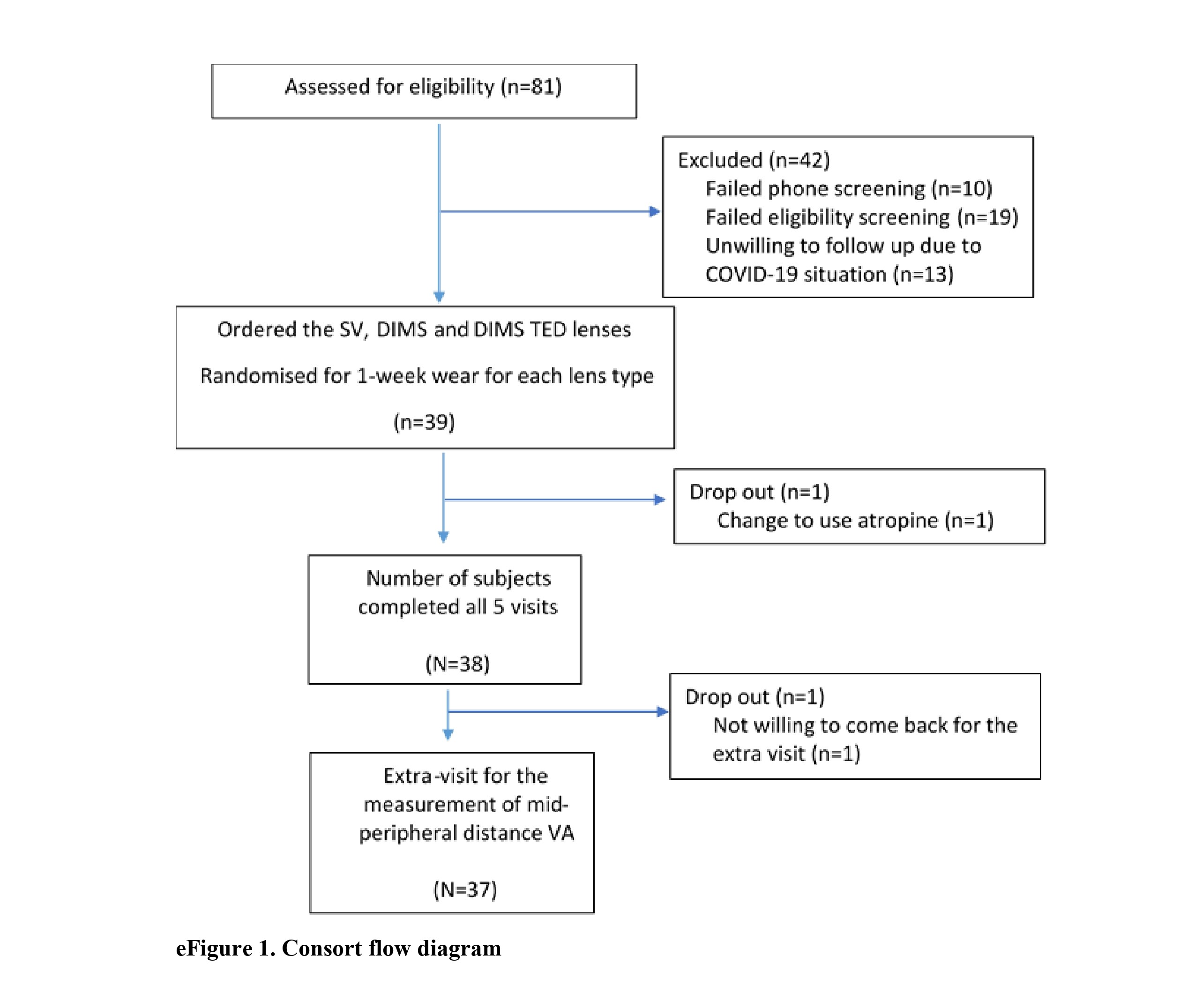

Supplement: Supplement 1 [file tvst-15-7-32_s001.jpg]

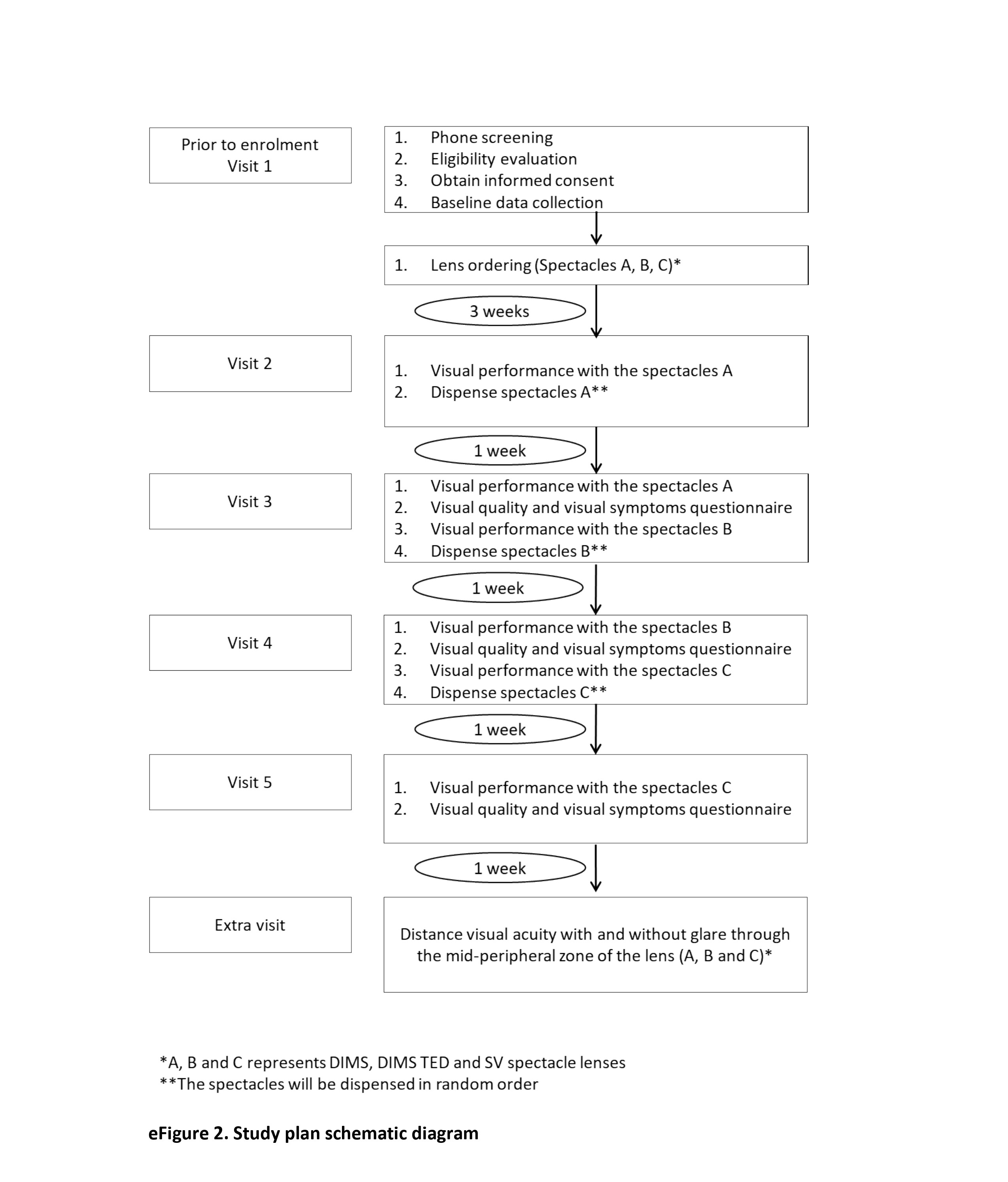

Supplement: Supplement 2 [file tvst-15-7-32_s002.jpg]

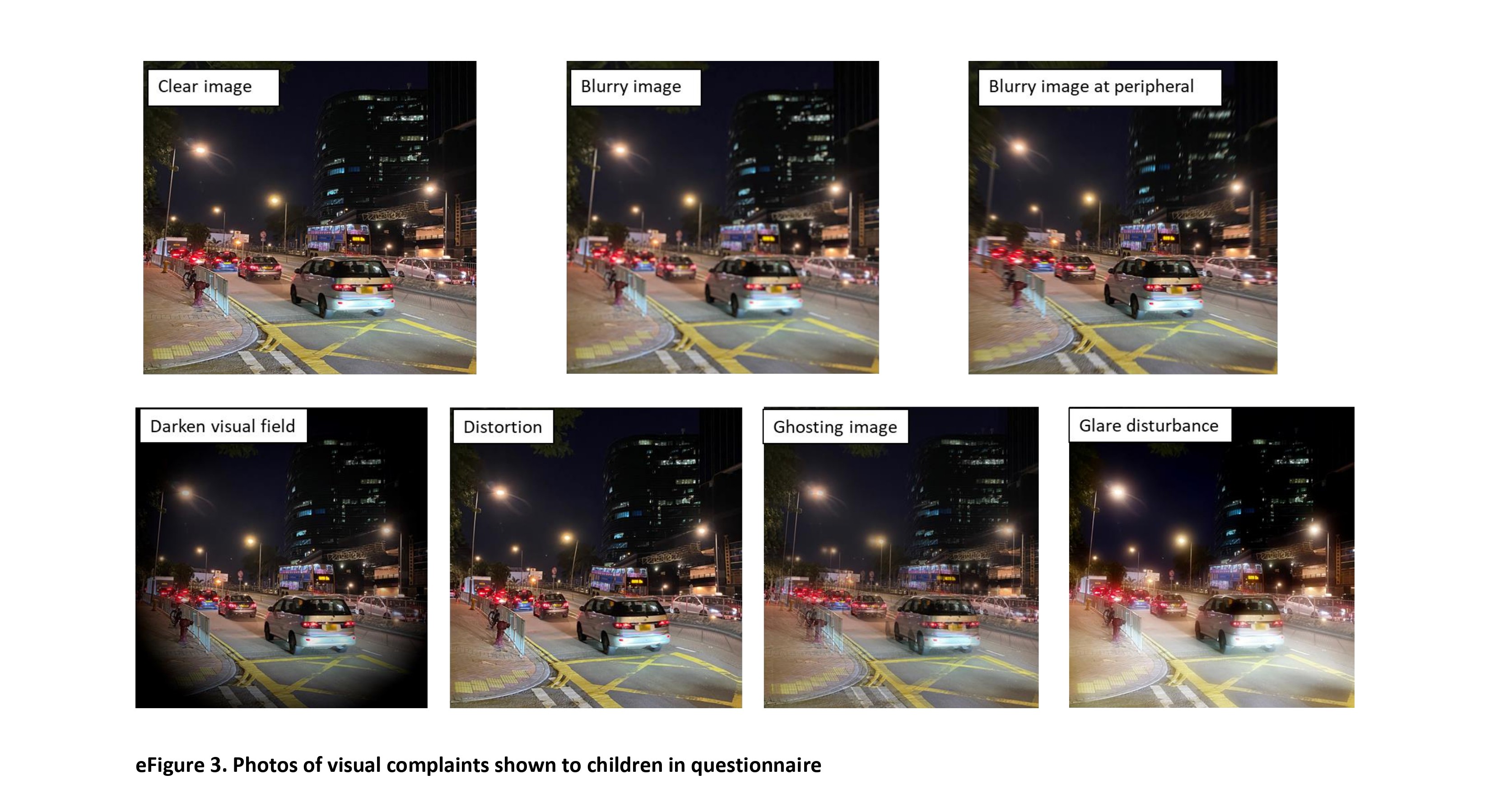

Supplement: Supplement 3 [file tvst-15-7-32_s003.jpg]

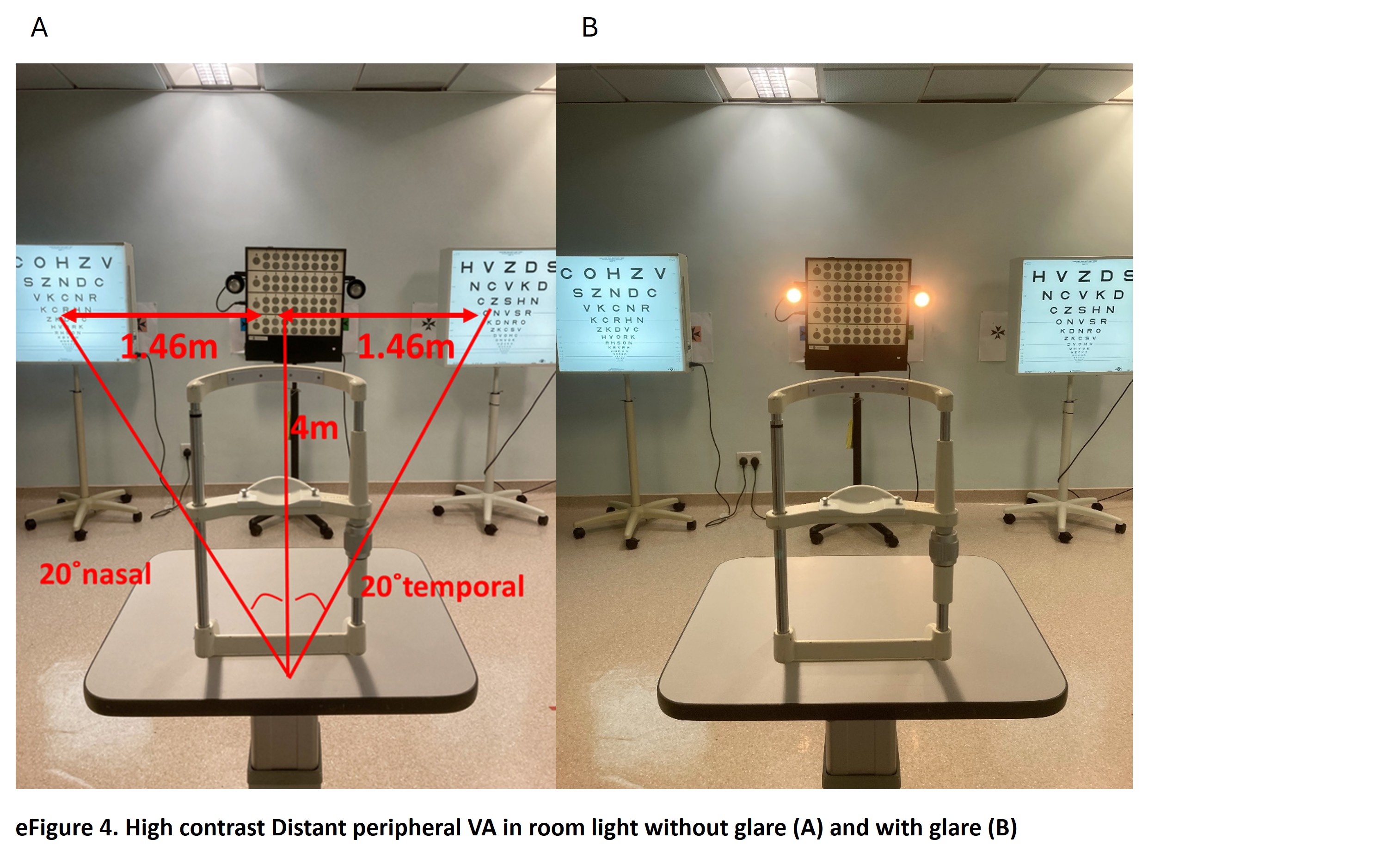

Supplement: Supplement 4 [file tvst-15-7-32_s004.jpg]
